# AMRI-59 functions as a radiosensitizer via peroxiredoxin I-targeted ROS accumulation and apoptotic cell death induction

**DOI:** 10.18632/oncotarget.23114

**Published:** 2017-12-09

**Authors:** Wan Gi Hong, Ju Yeon Kim, Jeong Hyun Cho, Sang-Gu Hwang, Jie-Young Song, EunAh Lee, Tong-Shin Chang, Hong-Duck Um, Jong Kuk Park

**Affiliations:** ^1^ Division of Applied Radiation Bioscience, Korea Institute of Radiological and Medical Sciences, Seoul, Korea; ^2^ Impedance Imaging Research Center, Kyung Hee University, Seoul, Korea; ^3^ College of Pharmacy, Ewha Womans University, Seoul, Korea

**Keywords:** peroxiredoxin, radiosensitizer, AMRI-59, ROS, non-small cell lung cancer

## Abstract

Previously, we identified AMRI-59 as a specific pharmaceutical inhibitor of peroxiredoxin (PRX) I enzyme activity. In this study, we examined whether AMRI-59 acts as a radiosensitizer in non-small cell lung cancer cells using clonogenic assays. The intracellular mechanisms underlying the radiosensitization effect of AMRI-59 were determined via immunoblotting in addition to measurement of ROS generation, mitochondrial potential and cell death. AMRI-59 activity *in vivo* was examined by co-treating nude mice with the compound and γ-ionizing radiation (IR), followed by measurement of tumor volumes and apoptosis. The dose enhancement ratios of 30 μM AMRI-59 in NCI-H460 and NCI-H1299 were 1.51 and 2.12, respectively. Combination of AMRI-59 with IR augmented ROS production and mitochondrial potential disruption via enhancement of PRX I oxidation, leading to increased expression of γH2AX, a DNA damage marker, and suppression of ERK phosphorylation, and finally, activation of caspase-3. Notably, inhibition of ROS production prevented ERK suppression, and blockage of ERK in combination with AMRI-59 and IR led to enhanced caspase-3 activation and apoptosis. In a xenograft assay using NCI-H460 and NCI-H1299, combined treatment with AMRI-59 and IR delayed tumor growth by 26.98 and 14.88 days, compared with controls, yielding enhancement factors of 1.73 and 1.37, respectively. Taken together, the results indicate that AMRI-59 functions as a PRX I-targeted radiosensitizer by inducing apoptosis through activation of the ROS/γH2AX/caspase pathway and suppression of ERK.

## INTRODUCTION

Lung cancer is one of the most lethal diseases worldwide. Non-small cell lung cancer (NSCLC), in particular, is associated with significantly low 5-year survival rates [1]. NSCLC therapy typically involves surgery, radiotherapy, and/or drug treatment. However, drug and radiation therapies, when used individually, frequently result in therapeutic resistance, which remains the primary treatment obstacle for most cancer types, including NSCLCs. Therefore, identification of novel therapeutic combinations of drugs and radiotherapy is an essential element of effective strategies to improve patient survival. Improving the effects of radiotherapy and minimizing damage to normal tissue entails modulation of different facets of cancer-specific processes, including DNA repair, cell cycle checkpoints, signal transduction, and the tumor microenvironment. For example, cancer cells with simultaneous impairment of DNA repair mechanisms and cell cycle checkpoints are unable to complete repair of damage caused by radiotherapy before cell death mechanisms are initiated [2]. The rationale for combining radiation and drug therapies is based on the concept that combinations of therapeutic modalities with different mechanisms of actions are more effective at targeting cancer than a single agent [3]. Although various conventional anticancer drugs have been employed as radiosensitizers, there remains an urgent need for new and more effective agents [2, 4]. Radiotherapy effects are classified into two categories, specifically, direct or indirect, whereby the direct effect is induction of DNA double- strand breakage and an indirect effect is the production of reactive oxygen/nitrogen species (ROS or RNS) resulting from ionization of various intracellular molecules, including H_2_O, by application of high-energy radiation [5-7].

Peroxiredoxin (PRX) acts as an intrinsic ROS scavenger that catalyzes reduction of H_2_O_2_ to water with a conserved cysteine residue serving as the site of oxidation by H_2_O_2_ [8]. 2-Cys PRXs are differentially distributed within cells. For example, PRX I and PRX II are located in the cytosol, PRX III in the mitochondria, and PRX IV in the endoplasmic reticulum and extracellular space. Several studies have reported overexpression of 2-Cys PRXs in various types of cancer cells and malignant tissues [9-12]. Therefore, inhibition of PRX presents an important strategy for effective radiosensitizer development. In an earlier study, we developed a novel screening system, which is a modified activity assay for mammalian PRX I using yeast Thioredoxin (yTrx) and yeast Thioredoxin Reductase (yTrxR) for inhibitors of PRX I, and then leading to the identification of AMRI-59 from small molecule libraries [13]. AMRI-59 has been characterized as an anti-cancer candidate as well as an inhibitor of PRX I via disruption of ROS, and deregulation of ROS is known to ultimately trigger cancer cell death through activation of both mitochondria- and apoptosis signal-regulated kinase-1-mediated signaling pathways. As AMRI-59 as well as radiotherapy treatment could induce cell death via disruption of intracellular ROS homeostasis, we investigated whether the potential of AMRI-59 as a novel radiosensitizer candidate that can enhance the cancer cell elimination efficacy of IR, both *in vitro* and *in vivo* in this study.

## RESULTS

### AMRI-59 acts as radiosensitizer by retarding cell growth *in vitro*

**Figure 1:**
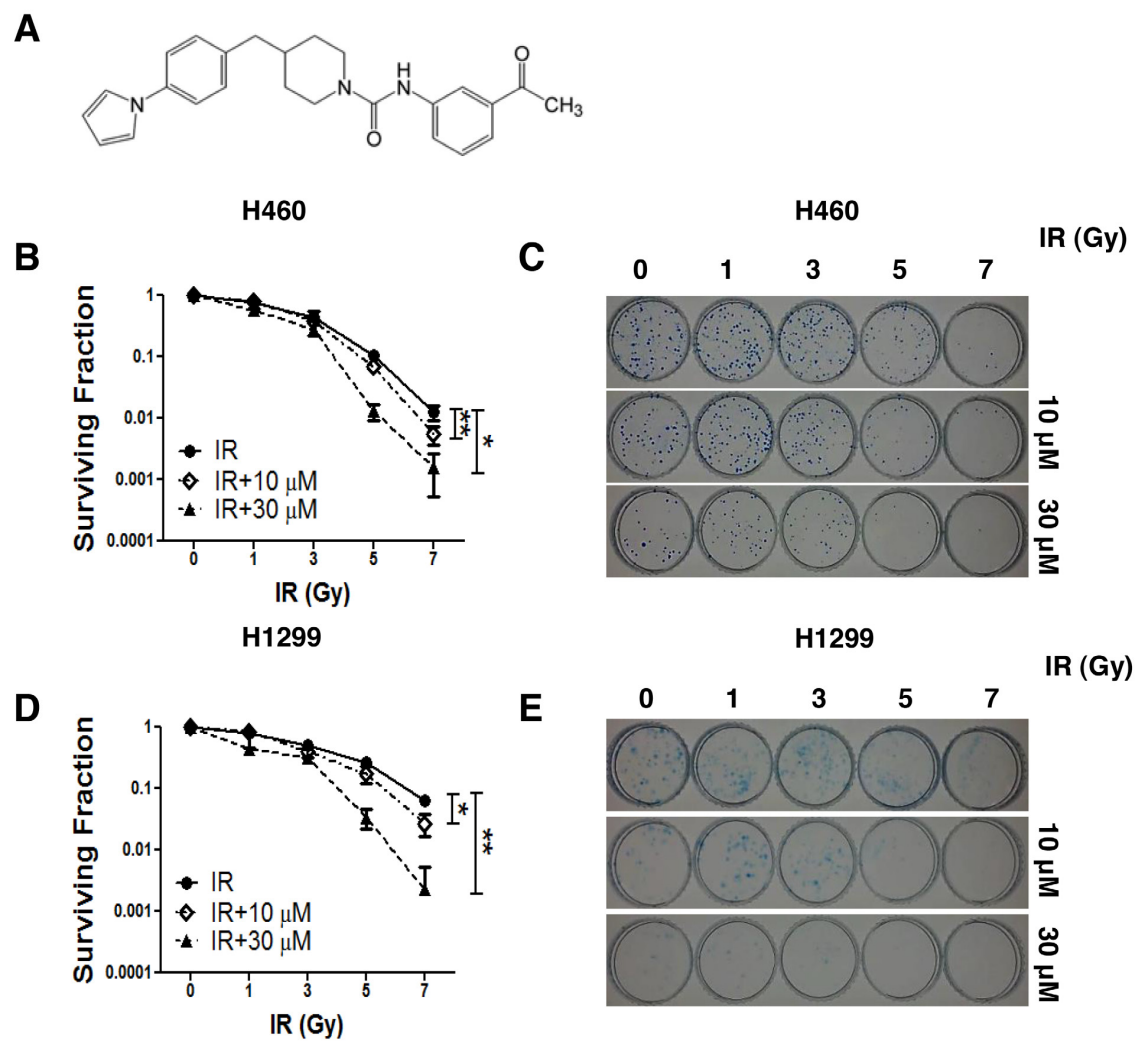
AMRI-59 induces retardation of NSCLC cell growth in conjunction with IR ‘IR’, radiation only; ‘IR+10 μM’ and ‘IR+30 μM’, combination of radiation with 10 μM and 30 μM AMRI-59, respectively. **(A)** Chemical structure of AMRI-59 (http://pubchem.ncbi.nlm.nih.gov/summary/summary.cgi?sid=93087). **(B-E)** Clonogenic assay for determining the radiosensitization effect of AMRI-59 against NCI-H460 (B, C) and NCI-H1299 (D, E). Representative data from experiments performed in triplicate are shown in each right panel.

We examined the radiosensitization effects of AMRI-59 (Figure 1A) on NCI-H460 and NCI-H1299 cell lines. The IC_50_ values of AMRI-59 in NCI-H460 and NCI-H1299 cells were previously determined as 4.5 and 21.9 μM, respectively [13]. Clonogenic assays with 10 or 30 μM AMRI-59 yielded a calculated dose enhancement ratio (DER) of 1.35 or 1.52 for NCI-H460 cells (Figure 1B, 1C; Table 1) and 1.35 or 1.83 for NCI-H1299 cells, respectively (Figure 1D, 1E; Table 1). Our data support a radiosensitization role of AMRI-59 in NSCLC cells.

**Table 1:** DER analysis

	DER (Dose-Enhancement Ratio)	
	10 μM	30 μM
**NCI-H460**	1.35	1.52
**NCI-H1299**	1.35	1.83

Note: DER (Dose-Enhancement Ratio) values were calculated from the clonogenic assay (Figure 1B, 1D). Radiation doses at a survival fraction of 0.1 of all groups (control, 10 μM and 30 μM AMRI-59 treatment groups for each NSCLC cell line) were acquired for NCI-H460 cells (control, 10 μM, 30 μM AMRI-59 treatment groups) as 5.1, 3.8 and 3.3 Gy and NCI-H1299 cells (control, 10 μM, 30 μM AMRI-59 treatment groups) as 5.9, 4.03 and 3.2 Gy, respectively. Using these doses, DER values were calculated as follows: radiation doses at a survival fraction of 0.1of the AMRI-59 treatment group/radiation doses at a survival fraction of 0.1of the IR-only treatment group.

### Combination of AMRI-59 and IR enhances caspase-3-mediated apoptosis

**Figure 2:**
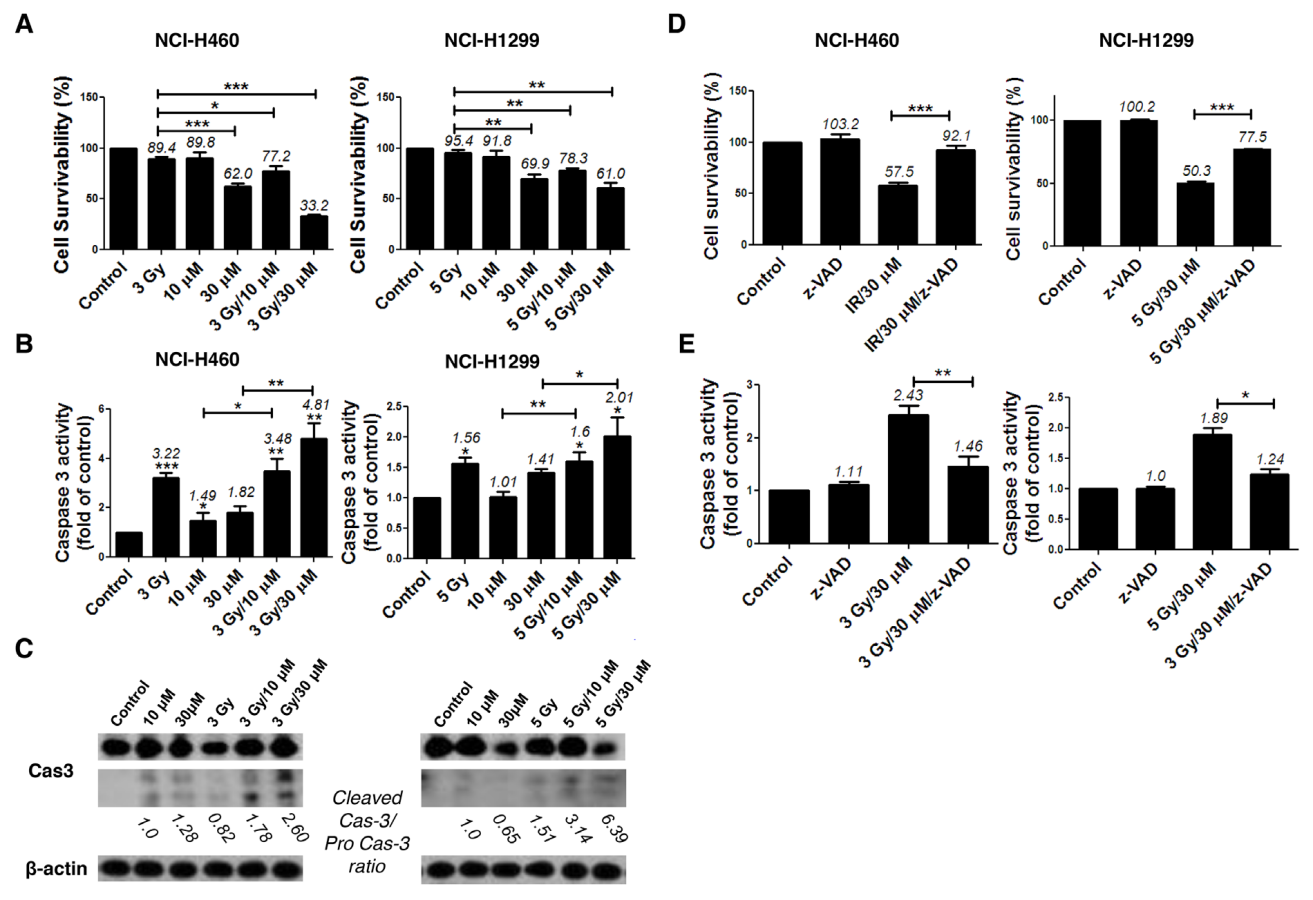
AMRI-59 induces NSCLC cell death and enhances apoptosis in conjunction with IR ‘Control’, mock control; ‘10 μM’ and ‘30 μM’, treatment with 10 and 30 μM AMRI-59 only; ‘3 Gy’ and ‘5 Gy’, treatment with 3 and 5 Gy IR only, respectively; ‘3 Gy/10 μM’, ‘3 Gy/30 μM’ and ‘5 Gy/10 μM’, ‘5 Gy/30 μM’, combination with 3 or 5 Gy IR and 10 or 30 μM AMRI-59, respectively; ‘z-VAD’, 20 μM of z-VAD-fmk pre-treatment. **(A)** PI uptake assay for detection of apoptosis. NCI-H460 or NCI-H1299 cells were treated with various doses of AMRI-59 and IR. **(B)** Caspase-3 activity detection with ELISA in NSCLCs treated with various doses of AMRI-59 and IR. **(C)** Immunoblot assay for detection of caspase-3 activation in cells treated with various doses of AMRI-59 and IR. ‘Pro-Cas3’ indicates pro-caspase-3 and ‘Cleaved Cas3’ is the cleaved form of caspase-3. **(D)** PI uptake assay and **(E)** Caspase-3 activity detection with or without pre-treatment of z-VAD-fmk for 1 h combined with AMRI-59 and IR. Representative data from experiments performed in triplicate are shown.

Next, we examined whether combined treatment with AMRI-59 and IR increases cell death to a greater extent than AMRI-59 or IR alone. Although AMRI-59 could induce growth retardation with various doses of IR in NCI-H460 and NCI-H1299 cells as shown in Figure 1, preliminary experiments on cell death to determine the optimal combination condition of AMRI-59 and IR indicated that 3 Gy IR+30 μM AMRI-59 for NCI-H460 and 5 Gy IR+30 μM AMRI-59 for NCI-H1299 cells were critical condition (data not shown). Firstly, propidium iodide (PI) uptake analyses were performed for quantitative assessment of cell death. Treatment with IR or AMRI-59 only did not induce a significant increase in apoptosis, compared with control, while the combination of AMRI-59 and IR enhanced cell death by at least >30% in both cell lines (Figure 2A and Supplementary Figure 1A). Immunoblot and caspase activity analyses were additionally performed to identify the cell death mechanisms, which disclosed increased cleavage as well as enhanced activity of caspase-3 (Figure 2B, 2C). Treatment with the pan-caspase inhibitor, z-VAD-fmk, attenuated the enhanced apoptosis and activity of caspase-3 induced by the combination (Figure 2D, 2E; Supplementary Figure 1B). These results imply that AMRI-59 and IR exert additive effects on apoptosis via activation of caspase-3. Therefore, the radiosensitization effect of AMRI-59 appears to originate from enhancement of apoptotic cell death through increased caspase-3 activity [14].

### Apoptosis induced by combined treatment with AMRI-59 and IR results from ROS production

**Figure 3:**
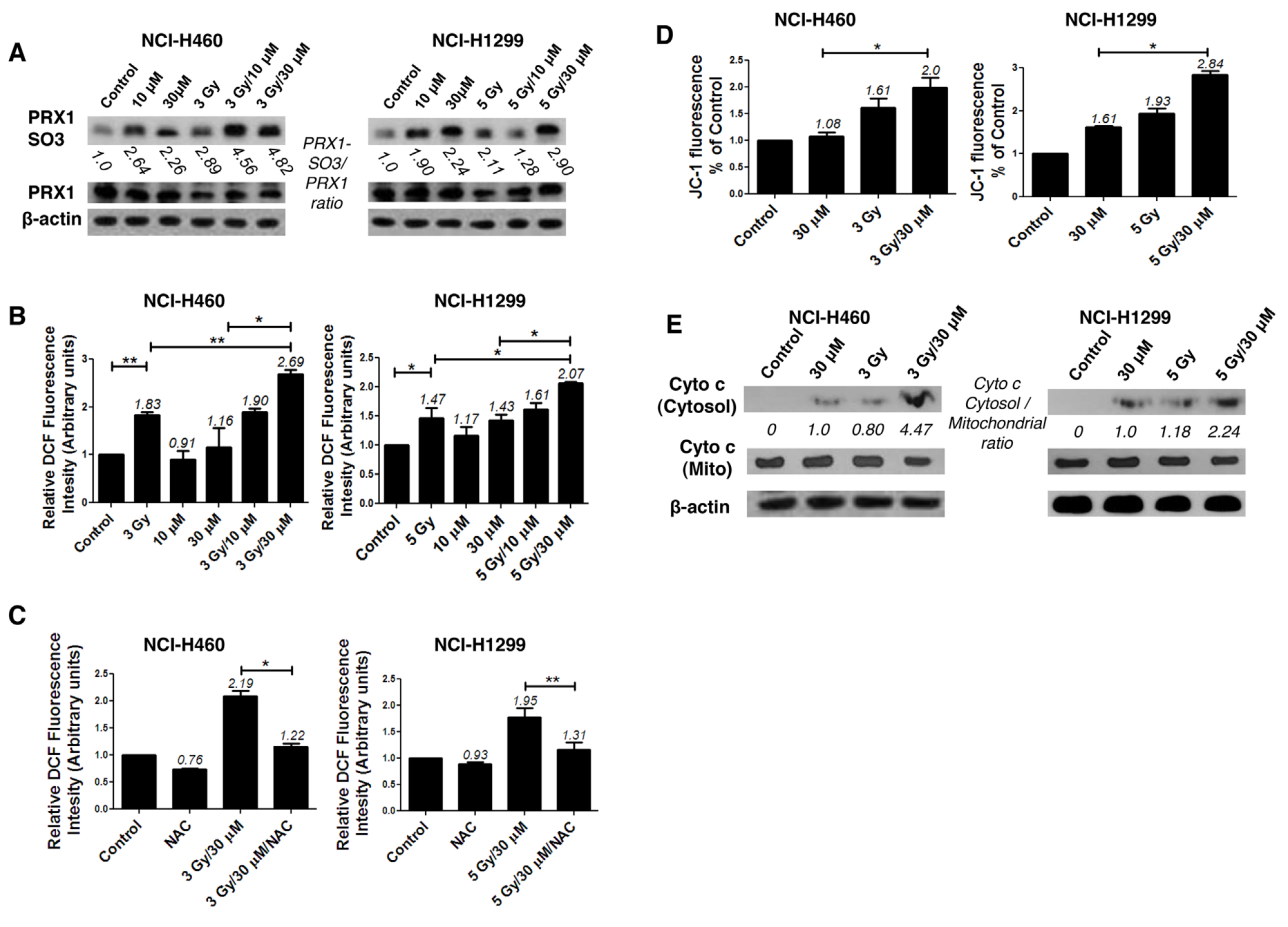
AMRI-59 induces ROS accumulation in conjunction with IR in NSCLC cells ‘Control’, mock control; ‘10 μM’ or ‘30 μM’, treatment with 10 or 30 μM AMRI-59 only; ‘3 Gy’ or ‘5 Gy’, treatment with 3 or 5 Gy IR only, respectively; ‘3 Gy/10 μM’, ‘3 Gy/30 μM’ and ‘5 Gy/10 μM’, ‘5 Gy/30 μM’, combination of 3 or 5 Gy IR and 10 or 30 μM AMRI-59, respectively; ‘NAC’, pre-treatment with 5 mM NAC. **(A)** Immunoblot analysis of oxidation of PRX I in in NCI-H460 and NCI-H1299 cells treated with a combination of AMRI-59 and IR **(B, C)** ROS determination. ROS detection with FACSorter in IR and AMRI-59-treated NSCLC cells (B), ROS detection with FACSorter in NAC pre-treated cells for 1 h treated with the IR and AMRI-59 combination (C). **(D)** Mitochondrial potential detection in NSCLC cells co-treated with IR and AMRI-59. **(E)** Immunoblot of cytochrome *c* release in NSCLC cells co-treated with IR and AMRI-59.

**Figure 4:**
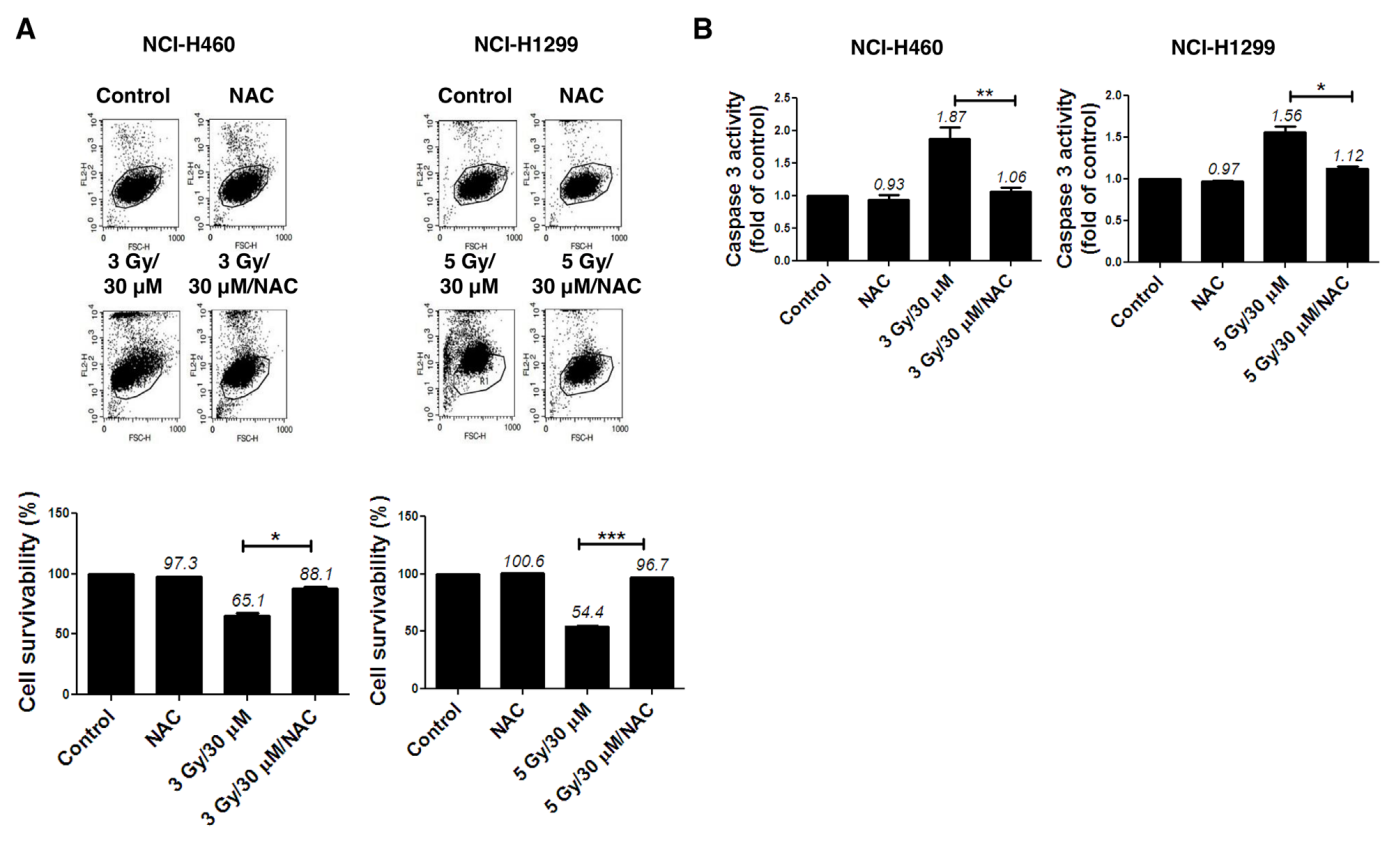
ROS production modulates apoptotic cell death in NSCLC cells subjected to combination of AMRI-59 and IR ‘Control’, mock control; ‘NAC’, pre-treatment with 5 mM NAC; ‘3 Gy/30 μM’ or ‘5 Gy/30 μM’ combination of 3 or 5 Gy IR and 30 μM AMRI-59; ‘3 Gy/30 μM/NAC’ or ‘5 Gy/30 μM/NAC’, combination of 3 or 5 Gy IR and 30 μM AMRI-59 with 5 mM NAC pre-treatment. **(A)** PI uptake assay for apoptotic cell death in the NAC-pre-treatment groups for 1 h subjected to AMRI-59 and IR. **(B)** Caspase-3 activity detection in cells with or without NAC pre-treatment for 1 h subjected to AMRI-59 and IR. Representative data from experiments performed in triplicate are shown.

The N-terminal conserved cysteine (Cys^52^-SH) of PRX I is selectively oxidized to cysteine sulfenic acid (Cys^52^-SOH), which reacts with Cys^173^-SH of the other subunit to form an intermolecular disulfide that is subsequently reduced by an appropriate electron donor. However, the sulfenic intermediate (Cys^52^-SOH) is easily over oxidized to cysteine sulfinic acid (Cys-SO_2_H) or cysteine sulfonic acid (Cys-SO_3_H) before it is able to form a disulfide under oxidative stress conditions [8]. Therefore, an increase in the cysteine sulfonic acid (Cys-SO_3_H) form of PRX I is suggestive of ROS induction. Consistent with our previous report that AMRI-59 stimulates Cys-SO_2_H formation [13], we observed that treatment with AMRI-59 or IR only generated the Cys-SO_3_H form of PRX I. Combination of AMRI-59 and IR synergistically induced an increase in the Cys-SO_3_H form of PRX I (Figure 3A) and ROS production (Figure 3B). Notably, treatment with NAC, a ROS scavenger, attenuated this increase in ROS production (Figure 3C; Supplementary Figure 2). Our results indicate that generation of the Cys-SO_3_H form of PRX I by the combination of AMRI and IR triggers an increase in ROS production. Mitochondrial membrane potential (*ΔΨ*_m_) of NSCLC cells was increased upon co-treatment with AMRI-59 and IR (Figure 3D), accompanied by cytochrome *c* release (Figure 3E). *ΔΨ*_m_ disruption followed by cytochrome *c* release is a well-established activation model of the caspase cascade. To evaluate the relationship between ROS production and apoptotic cell death, cells were pre-treated with NAC before combined treatment with AMRI-59 and IR. Interestingly, blockage of ROS production inhibited caspase-3 activation and cell death almost perfectly (Figure 4A, 4B). Our results collectively indicate that PRX I inhibition by AMRI-59 further contributes to IR-induced ROS production, resulting in caspase-activated apoptosis.

### Blockage of ROS production compensates for the DNA damage effect of AMRI-59

**Figure 5:**
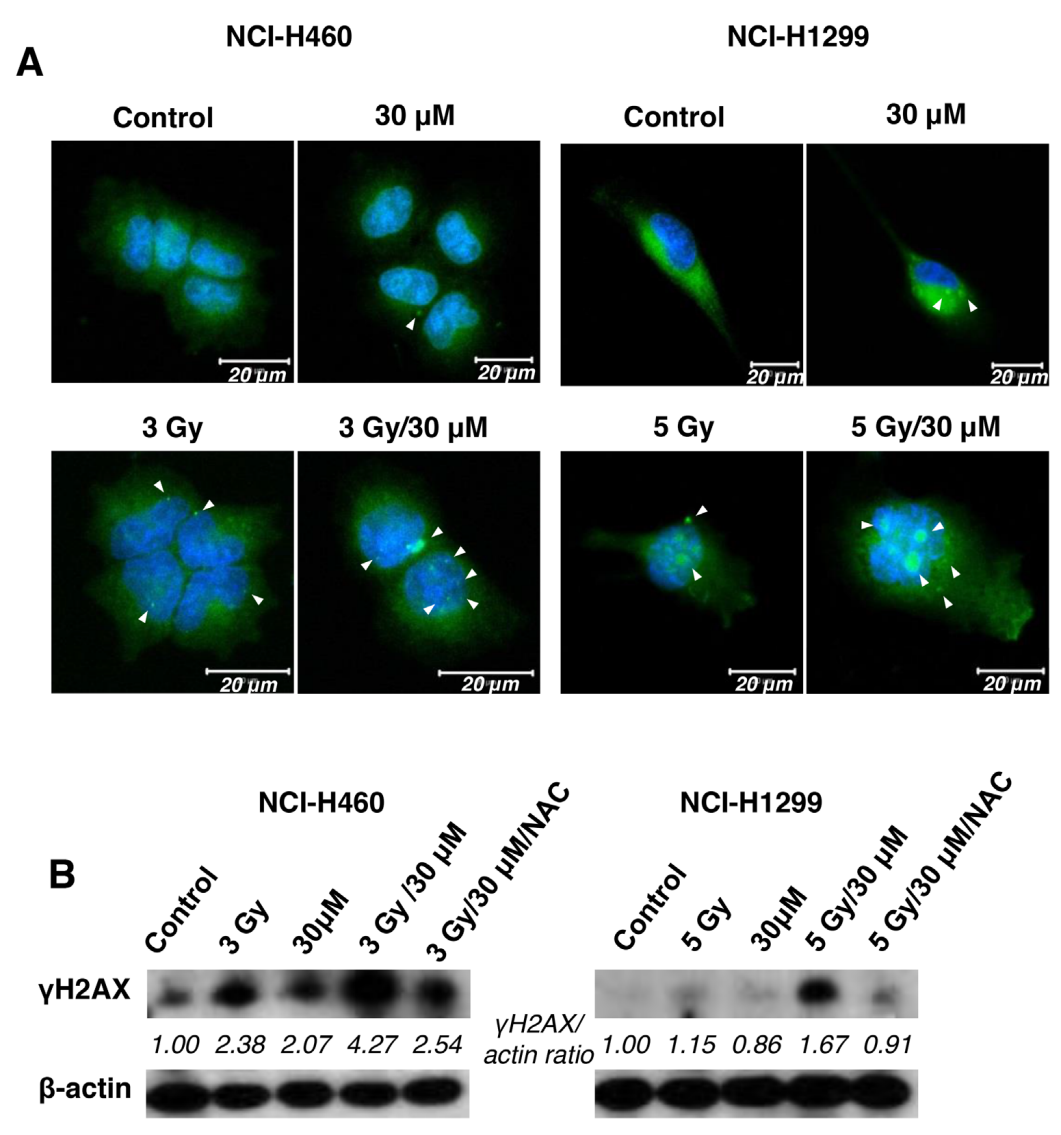
The combination of AMRI-59 and IR induces enhanced DNA damage ‘Control’, mock control; ‘30 μM’, 30 μM AMRI-59 only; ‘3 Gy’, or ‘5 Gy’, 3 or 5 Gy IR only, respectively; ‘3 Gy/30 μM’ or, ‘5 Gy/30 μM’ combination of 3 or 5 Gy IR and 30 μM AMRI-59. **(A)** Immunohistochemical assay showing formation of γH2AX foci (*arrow*) in IR only, AMRI-59 only and combined AMRI-59 and IR groups. White bars in each image indicate 20 μM. **(B)** Immunoblot assay for detection of elevated γH2AX in IR only, AMRI-59 only and combined AMRI-59 and IR groups. Representative data from experiments performed in triplicate are shown.

As DNA breakage in cells is a one of main targets of IR-induced cell death, we further examined whether the combination of AMRI-59 and IR stimulates DNA damage. To detect DNA damage, we performed IHC and immunoblot assays with the anti-γ H2AX antibody. H2AX, histone family member X, is one of several genes encoding histone H2A. The protein contributes to the structure of the DNA chromosome by facilitating nucleosome formation. During IR-induced DNA double-strand break (DSB) formation, H2AX undergoes phosphorylation at serine 139 due to the kinase activity of ATM or DNA-PKcs, producing γ-H2AX [15, 16]. As shown in Figure 5A, co-treatment with AMRI-59 and IR produced increased foci of γ-H2AX relative to AMRI-59 or IR only in both NCI-H460 and NCI-H1299 cells, as observed with the IHC assay. Data from the immunoblot assay consistently revealed greater γ-H2AX expression with the combination of AMRI-59 and IR. NAC treatment led to a decrease in γ-H2AX expression (Figure 5B). These results imply that AMRI-59 and IR synergistically induce greater DNA damage via promotion of ROS production.

### Elimination of ERK is involved in the radiosensitizing effect of AMRI-59

**Figure 6:**
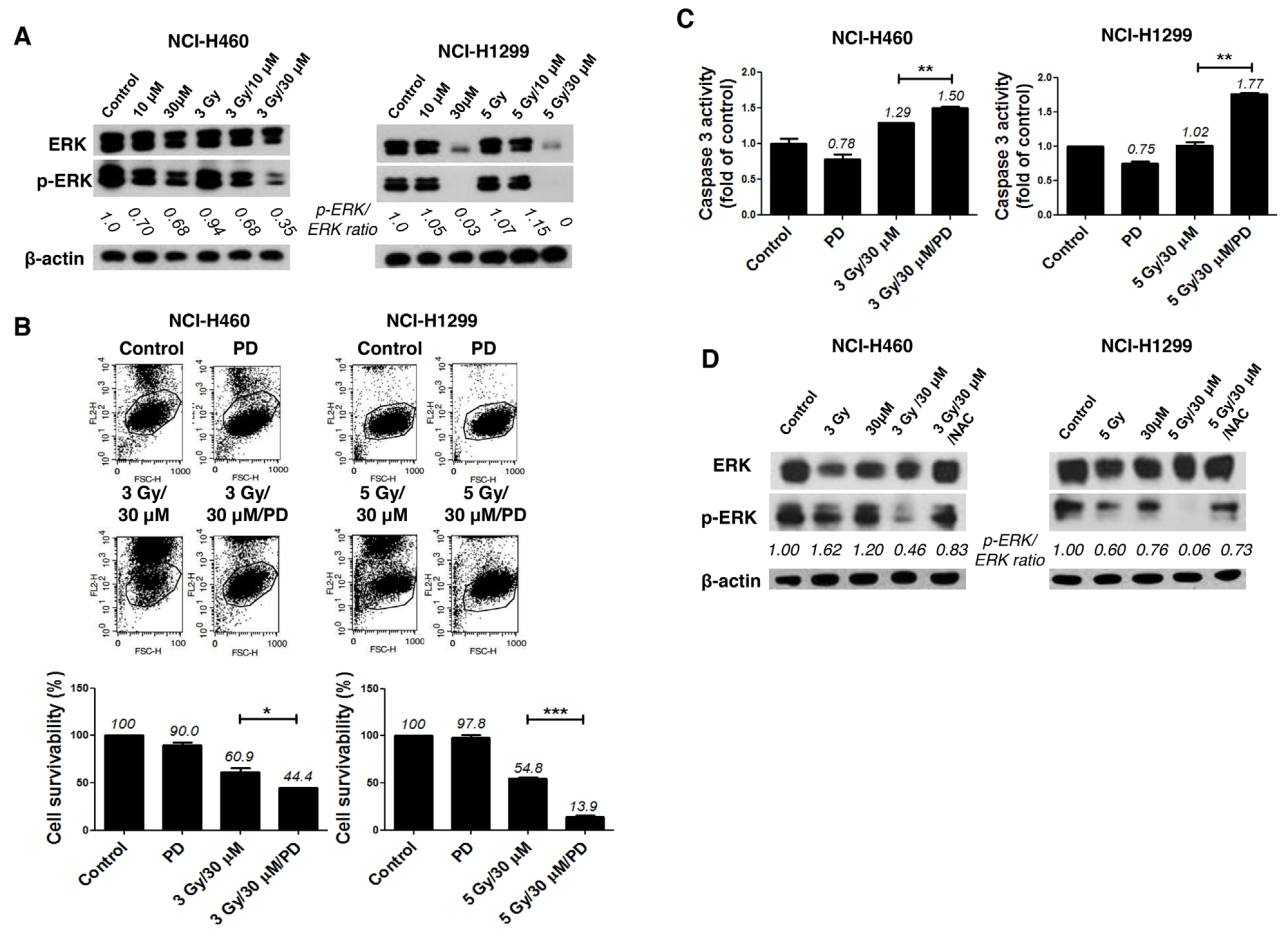
Combination of AMRI-59 and IR promotes apoptotic cell death via elimination of ERK activity ‘Control’, mock control; ‘3 Gy’ or ‘5 Gy’, 3 or 5 Gy IR only, respectively; ‘10 μM’ or ‘30 μM’, treatment with 10 or 30 μM AMRI-59 only; ‘3 Gy/30 μM’ or ‘5 Gy/30 μM’ combination of 3 or 5 Gy IR and 30 μM AMRI-59; ‘3 Gy/30 μM/PD’ or ‘5 Gy/30 μM/PD’, combination of 3 or 5 Gy IR and 30 μM AMRI-59 with 10 μM PD98059 pre-treatment. ‘PD’, 10 μM PD98059 pre-treatment only. **(A)** Immunoblot assay for detection of phosphorylated and basal ERK. **(B)** PI uptake assay for detection of apoptotic death in cells with or without PD98059 pre-treatment for 1 h. **(C)** Caspase-3 activity detection in cells subjected to AMRI-59 and IR with or without PD pre-treatment for 1 h. **(D)** Immunoblot assay for detection of phosphorylated and basal ERK with NAC pre-treatment for 1 h. Representative data from experiments performed in triplicate are shown.

To elucidate the intracellular signaling mechanisms underlying the radiosensitizing effect of AMRI-59, we examined whether the combination of AMRI-59 and IR modulates activities of proteins in the mitogen-activated protein kinase (MAPK) family, including p38, ERK and c-Jun N-terminal kinase (JNK), by examining their phosphorylation status in NCI-H460 and NCI-H1299 cells via immunoblot analysis. Co-treatment with AMRI-59 and IR suppressed basal protein as well as phosphorylation levels of ERK (Figure 6A) while inducing no changes in JNK and p38 phosphorylation (data not shown). Pre-treatment with PD98059 (PD), a chemical inhibitor of ERK, enhanced cell death induced by the combination of AMRI-59 and IR (Figure 6B) and enhanced caspase-3 activation (Figure 6C). Pre-treatment with PD increased apoptosis and caspase-3 activation in cells treated with the combination of AMRI-59 and IR by more than 2-fold, compared with cells subjected to the combination without PD pre-treatment. Suppression of ERK by the AMRI-59 and IR combination was attenuated upon NAC pre-treatment (Figure 6D), indicating that ROS directly disrupts both ERK protein expression and protein activity. In NSCLC, the Ras/Raf/MEK/ERK pathway plays diverse physiological roles in pathogenesis, progression, and malignancy behaviors [17, 18]. IR activates ERK, resulting in endothelial radiosensitization, which considered one of the primary determinants of tumor radiosensitivity. These reports suggest that inhibition of ERK presents an attractive strategy for sensitizing tumor cells to radiotherapy [19-21].

### AMRI-59 suppresses CREB-1 activity to induce radiosensitization

**Figure 7:**
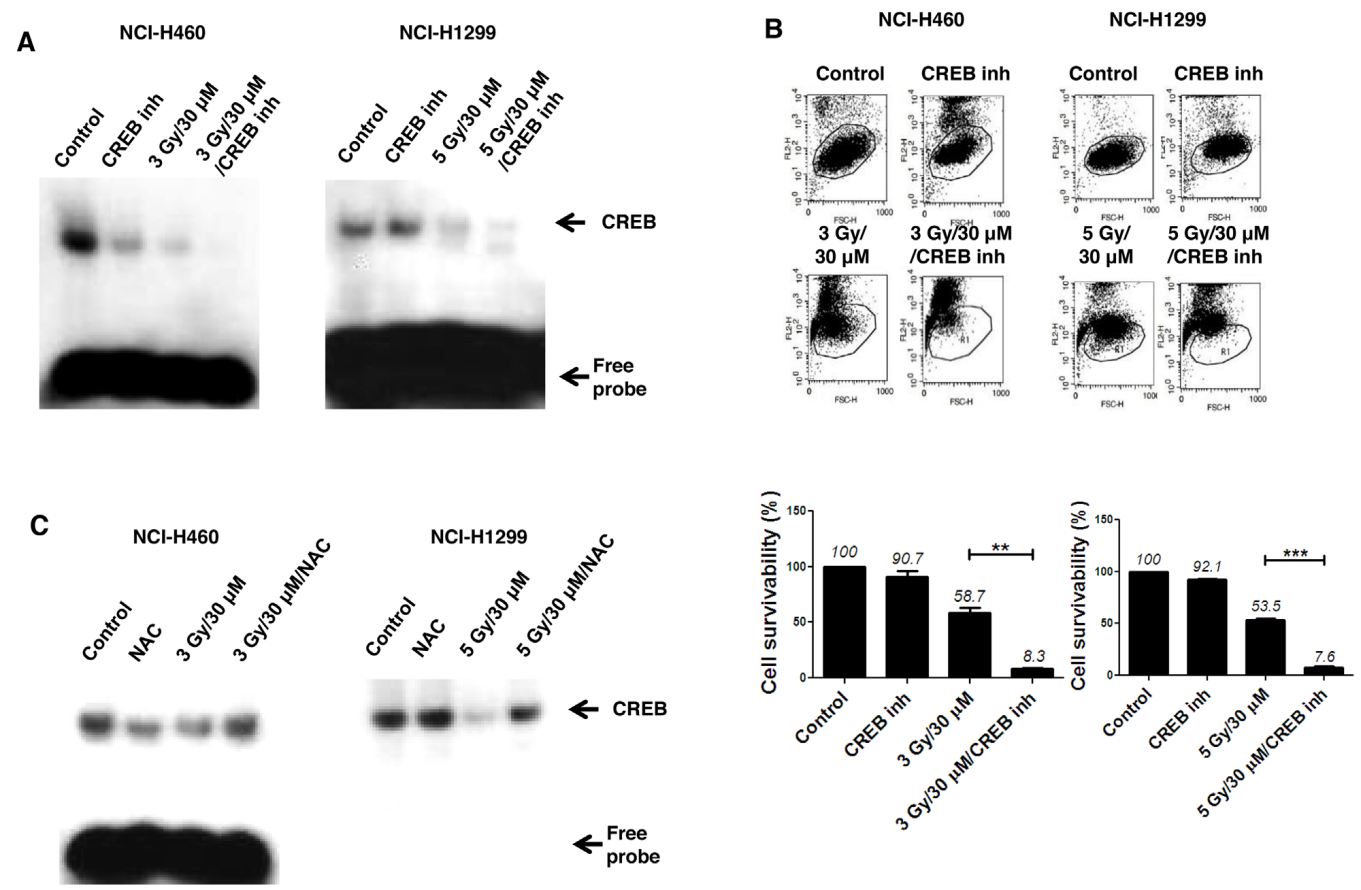
Attenuation of CREB-1 activity is a step in the apoptotic cell death pathway triggered by the combination of AMRI-59 and IR ‘Control’, mock control; ‘CREB inh’, pre-treatment with 10 μM CREB-1 inhibitor only; ‘NAC’, pre-treatment with 5 mM NAC; ‘3 Gy/30 μM’ or ‘5 Gy/30 μM’, combination of 3 or 5 Gy IR and 30 μM AMRI-59; ‘3 Gy/30 μM/NAC’ or ‘5 Gy/30 μM/NAC’, combination of 3 or 5 Gy IR and 30 μM AMRI-59 with 5 mM NAC pre-treatment; ‘3 Gy/30 μM/CREB inh’ or ‘5 Gy/30 μM/CREB inh’, combination of 3 or 5 Gy IR and 30 μM AMRI-59 with 10 μM CREB inhibitor pre-treatment. **(A)** EMSA assay for detection of activated CREB-1 in cells subjected to AMRI-59 and IR with or without CREB inhibitor pre-treatment for 1 h. **(B)** PI uptake assay for detection of apoptotic death in cells treated with a combination of AMRI-59 and IR with or without CREB-1 inhibitor pre-treatment. **(C)** EMSA assay for detection of activated CREB-1 in cells treated with a combination of AMRI-59 and IR treatment with or without NAC pre-treatment for 1 h. Representative data from experiments performed in triplicate are shown.

To address the possible involvement of transcription factors, we performed electrophoretic mobility shift assay (EMSA) using several radiolabeled probes, including AP1, OCT1, TFIID, NF-κB and CREB-1. CREB-1 was subsequently identified as a major transcription factor involved in the radio sensitization effect of AMRI-59 against NSCLC cells (Figure 7A). Moreover, the combined effects of AMRI-59 and IR were amplified with a CREB-1 chemical inhibitor, resulting in further promotion of cell death (Figure 7A, 7B). NAC treatment led to recovery of CREB-1 activity in both NSCLC cell lines, indicating that suppression of CREB-1 activation may be controlled by ROS production.

In a previous study, we demonstrated that IR promotes phosphorylation of CREB-1 at serine 133. Specifically, the transcriptional activity of CREB-1 was shown to be enhanced by interactions with CREB-binding protein (CBP) and CREB-1 identified as one of the potential molecular targets for radiosensitization [22-24]. The data collectively suggest that ERK and CREB-1 are signaling mediators of the radiosensitization effects of AMRI-59 that act through regulation of caspase-3 activation to promote apoptotic cell death in response to elevated ROS production.

### *In vivo* radiosensitization effect of AMRI-59

**Figure 8:**
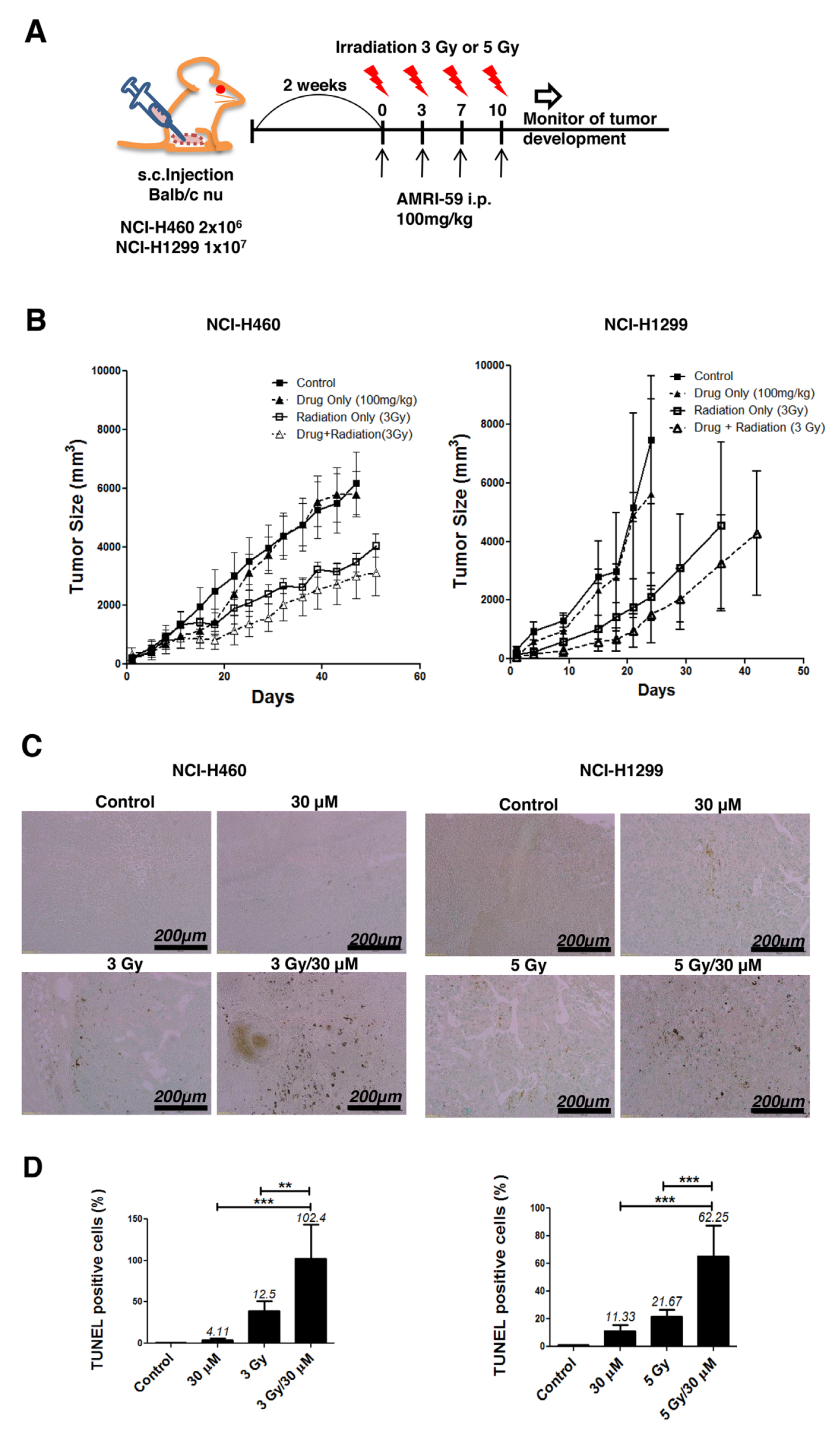
Combination of AMRI-59 and IR enhances apoptotic cell death *in vivo* ‘Control’, mock control; ‘Drug only’, 100 mg/kg AMRI-59 only; ‘Radiation only’, Treatment of NCI-H460 or NCI-H1299 with 3 or 5 Gy IR only; ‘Drug/Radiation’, combination of 3 or 5 Gy IR and 100 mg/kg AMRI-59 for treatment of NCI-H460 or NCI-H1299, respectively. **(A)** Images indicate schedules for *in vivo* experiments. Mice were injected with NCI-H460 (2 x 10^6^) or NCI-H1299 (1 x 10^7^) cells and divided into four treatment groups (5 mice/group). **(B)** Calculation of xenograft sizes. **(C)** TUNEL assay with dark brown dots considered TUNEL-positive cells. **(D)** Quantitative analysis of TUNEL-positive cells. Representative data from experiments performed in triplicate are shown.

**Figure 9:**
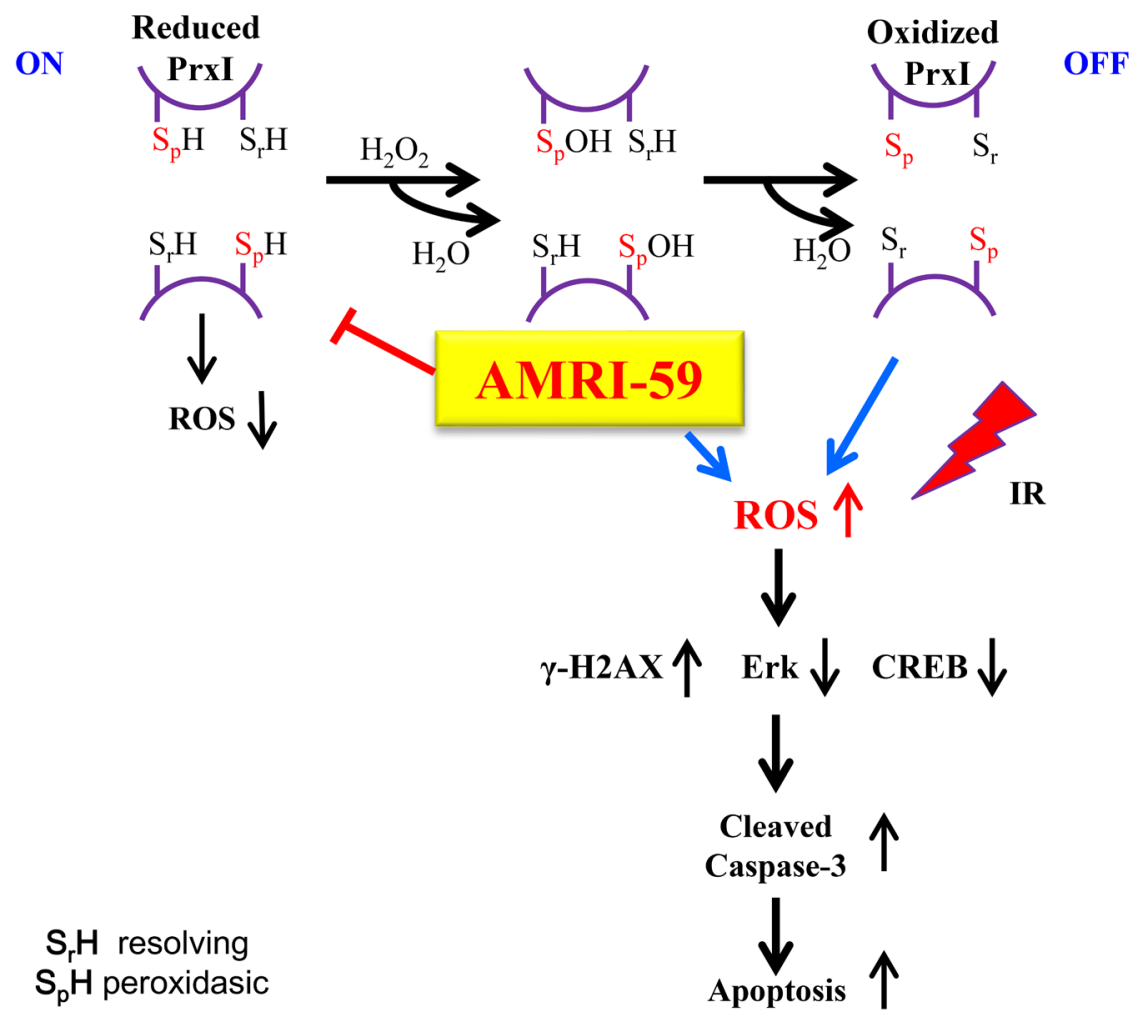
Scheme of the intracellular mechanism of the radiosensitization effect of AMRI-59 The N-terminal conserved cysteine (Cys^52^-SH) of PRX I was selectively oxidized by H_2_O_2_ to cysteine sulfenic acid (Cys^52^-SOH), which then reacted with Cys^173^-SH of the other subunit to form an intermolecular disulfide that was subsequently reduced by an appropriate electron donor. AMRI-59 disrupts the oxidation stage of catalytic site Cys-SH by H_2_O_2,_ and result to accumulation of intracellular ROS. In this study, IR treatment also promotes ROS production, and then induces synergic ROS increase. The increased ROS promotes increase of γ-H2AX level and decrease of ERK and CREB-1 activation, which are ones of main components of cell survival signaling. Suppression of cell survival signaling promotes apoptotic cell death resulted from caspase-3 activation.

On the basis of *in vitro* results, we further examined the radiosensitizing effects of AMRI-59 *in vivo* by constructing xenografts with NCI-H460 or NCI-H1299 cells, as described in Materials and methods (Figure 8A). Assessment of the time required to reach a tumor volume of 3,000 mm^3^ showed that the combination treatment produced an 26.98- or 14.88-day growth delay, compared with control xenografts derived from both cell lines, respectively, a difference that translated into an enhancement factor of 1.73 and 1.37, respectively (Figure 8B; Table 2). These results clearly indicate that AMRI-59 acts as a radiosensitizer *in vivo* as well as *in vitro*. A TUNEL assay performed on tumor tissue revealed that the number of apoptotic cells in the combination group, expressed as a percentage of the total cell population, was about 8.2- and 2.9-fold higher than that in the IR-only group, and 25- and 6-fold higher than that in the AMRI-59-only group of NCI-H460 and NCI-H1299 cell-derived xenografts, respectively (Figure 8C, 8D). Our results collectively demonstrate that the combination of AMRI-59 and IR exerts an enhanced radiosensitizing effect through increased induction of apoptosis, both *in vivo* and *in vitro* Figure 9.

**Table 2:** Tumor growth delay analysis

	NCI-H460		NCI-H1299	
Treatment	Days	Growth delay	Days	Growth delay
**Control**	21.90		18.18	
**AMRI-59-only**	23.95	2.05	19.44	1.26
**IR-only**	36.27	14.37	28.14	9.96
**Combination**	48.88	26.98	33.06	14.88
**Enhancement factor**	1.73		1.37	

Note: AMRI-59: PRX I inhibitor. Tumor xenograft volumes were measured and the radiosensitizer effect of AMRI-59 analyzed *in vivo*. ‘Days’ indicates the period required for xenografts in each group to reach 3000 mm^3^; ‘growth delay’ indicates the additional period required for xenografts in each group to reach 3000 mm^3^ beyond that required for tumors in the control group to reach the same volume. The enhancement factor was calculated as follows: (growth delay of combination-treated tumors - growth delay of tumors treated with AMRI-59 only)/growth delay of tumors treated with IR only.

## DISCUSSION

Further to our initial identification of AMRI-59 as a potent PRX I inhibitor that suppresses cellular ROS levels, we examined its potential radiosensitizing effects *in vitro* and *in vivo* in this study. A combination of AMRI-59 and IR induced growth retardation of NSCLC cells (Figure 1) due to enhanced apoptosis, as shown in Figure 2. Apoptosis occurs via two major apoptotic pathways, specifically, intrinsic and extrinsic. The intrinsic pathway is activated by external stress and leads to changes in mitochondrial permeability and stimulation of caspase-9 activity. The extrinsic pathway begins with death receptor/ligand binding and proceeds through caspase-8 activation. Both apoptotic pathways engage caspases, starting with an ‘initiator’ caspase (caspase-8 and -9 for the extrinsic and intrinsic pathway, respectively) and culminate with activation of caspase-3, a common ‘executioner’ caspase [14]. Consistent with the reported involvement of these pathways, we found that combination treatment with AMRI-59 and IR promoted the activity and cleavage of the common executioner caspase (caspase-3) and inhibition of caspase-3 activation prevented apoptosis *in vitro*. Since the ultimate purpose of radiotherapy is to eradicate cancer, typically by activating apoptosis mechanisms (including a number of apoptosis-related proteins such as p53, p21, Bcl-2, Bax and caspases), apoptotic machinery is the subject of intensive research to develop effective therapeutic tools for enhancing the effects of radiotherapy [25-28]. Consistently, combined therapy with AMRI-59 and IR enhanced apoptotic cell death as well as tumor growth retardation in an animal model, as shown in Figure 8. Based on these findings, we tentatively hypothesized that AMRI-59 acts as a radiosensitizer and further investigated the intracellular machinery underlying its effects on tumor cells.

Next, we showed that the radiosensitizer activity of AMRI-59 resulted from increased oxidation of PRX (Figure 3A), which in turn enhanced ROS production, mitochondrial potential, and ultimately cytochrome *c* release (Figure 3). Data from our previous study showed that AMRI-59 treatment triggers an increase in the oxidized form of PRX [13]. Combination with IR treatment further promoted this PRX oxidation, resulting in augmented ROS production. ROS production is one of major effects of IR irradiation that can induce two major types of cellular damage, either direct or indirect. The representative direct effect of IR at the cell level is disruption of genetic materials, including DNA double strand breakage, while the indirect effect involves free radical production resulting from water radiolysis in the cytoplasm [29]. The energy absorption from IR treatment of water results in both excitation and ionization of the molecule, leading to the production of free radicals, such as ROS (O_2_^·−^, ^·^OH, and H_2_O_2_) and reactive nitrogen species (RNS; ^·^NO and ONOO^−^), which may, in turn, attack other critical intracellular molecules, including lipids, thiols, proteins and DNA bases [30-32].

Many cancer types are associated with elevated basal levels of ROS resulting from cell transformation into malignancy, which induced by multiple physiological changes, including activation of specific oncogenes. Intracellular ROS serve as essential signaling molecules that regulate numerous cellular processes enhancing proliferation, migration, and other malignant phenotypes [33]. Under excess oxidative stress conditions, many types of cancer cells acquire adaptive mechanisms of resistance. As mammalian cells have well-defined endogenous antioxidant enzymes, such as superoxide dismutases, catalase, glutathione peroxidases (GPx) and PRXs, one mechanism of adaptation is to increase the expression of these proteins [34, 35]. Overexpression of cytosolic PRX I has been reported in various human cancer cell and tissue types [9-12], and its enhanced expression shown to contribute to the development of cisplatin resistance in ovarian and breast cancer [36]. Moreover, previous studies have demonstrated that elimination of PRX I enzyme promotes lung cancer cell susceptibility to IR-induced cell death [37, 38]. Although radiosensitizer effect of AMRI-59 did not compared to those of cisplatin or carboplatin that have been used as radiosensitizer generally [39], we identified AMRI-59, PRX I-targeted inhibitor, could be used as radiosensitizer against NSCLC cells *in vivo* and *in vitro*. Therefore, the collective findings support the utility of PRX I as a potential target for the development of anticancer drugs.

Furthermore, disruption in the ROS balance induced by the combination of AMRI-59 and IR evoked enhanced apoptotic cell death, accompanied by DNA damage and suppression of ERK and CREB-1 activation. NAC treatment reversed the combined effects of AMRI-59 and IR, suggesting that that these three phenomena occur downstream of ROS production. In particular, ERK and CREB-1 may be important mediators of the radiosensitization effects of various regents, including AMRI-59, since we previously identified these molecules in NSCLSC cells as the main targets for suppression by several radiosensitizers [22, 40]. To improve efficiency of radiotherapy, radiosensitizer development focus to increase the effect on the tumour or to decrease the side-effects on normal tissues. Two research fields have to accomplish for safe and efficient radiosensitizer development; first, a knowledge of the molecular response of cells and tissues to IR, and second, a new excavation of the exploitable genetic alterations in tumours [41]. Although we did not determine whether AMRI-59 protects normal tissue from IR-induced damage or whole cell death is mediated by the ROS/caspase pathway in response to the AMRI-59/IR combination, data from the current study support a novel role of AMRI-59 as a radiosensitizer that promotes apoptotic death of NSCLC cells through activation of ROS-induced apoptosis and suppression of ERK/CREB-1 activities. Our findings provide novel insights that support the development of PRX-targeted inhibitors as radiosensitizers and further exploration of ERK and CREB-1 as potential targets for therapeutic agents against NSCLC.

## MATERIALS AND METHODS

### Cell culture and chemical reagents

The human NSCLC cell lines, NCI-H460 and NCI-H1299, were purchased from American Type Culture Collection (Rockville, MD, USA). PD98059, N-acetyl-L-cysteine (NAC), 2’,7’-dichlorofluorescin diacetate (DCF-DA), N-(4-Chlorophenyl)-3-hydroxy-2-naphthamide (CBP-CREB Interaction Inhibitor; CREB-1 inhibitor) and N-benzyloxycarbonyl-Val-Ala-Asp-fluoromethylketone (z-VAD-fmk) fluoromethylketone fluoromethylketone fluoromethylketone were fluoromethylketoneobtained from Calbiochem (La Jolla, CA, USA). AMRI-59 [N- (3-acetylphenyl)-4- (biphenyl-4- ylmethyl) piperidine-1-carboxamide] was acquired from AMRI (Seoul, Korea). fluoromethylketone

### Clonogenic assay

Clonogenic assays were performed as described previously [42]. NCI-H460 and NCI-H1299 cells were seeded in 60 mm dishes in triplicate at densities estimated to yield 20-100 colonies/dish (100, 200, 400, 600 and 1000 cells/dish). After 24 h incubation, cells were pre-incubated with or without 10 or 30 μM AMRI-59 for 16 h and exposed to different doses of IR (1, 3, 5, 7 Grey [Gy]) using ^137^Cs as the radiation source (Atomic Energy of Canada, Ltd., Ontario, Canada). Treated and control cells were cultured for 10-14 days, and colonies >200 μm in diameter counted using a colony counter (Imaging Products, VA, USA). Dose enhancement ratio (DER) values were calculated with Excel program (Microsoft Co. USA) as describe in Table 1.

### Immunoblot analysis

Immunoblot experiments were performed as described previously [43]. Membranes were probed with antibodies against caspase-3, phosphorylated ERK (extracellular signal-regulated kinase), γH2AX, cytochrome *c* and total ERK (Cell Signaling Technology, Inc., Beverly, MA). Anti-PRX-SO_3_ and anti-PRX I antibodies were purchased from Abclone (Seoul, Korea). An anti-β-actin antibody (Sigma-Aldrich, St. Louis, MO) was used as the control for equal loading. Relative band densities of targets, determined densitometrically and normalized to that of β-actin or ERK, were analyzed using Image J software (NIH, USA).

### ROS detection assay

ROS production was detected as described previously [44]. Cells were seeded at a density of 1.5 × 10^5^ and subjected to various conditions. NCI-H460 cells or NCI-H1299 cells were pre-treated with 30 μM AMRI-59 for 6 h and then irradiated 3 or 5 Gy IR, respectively. After 48 h incubation, treated cells were incubated with 20 μM DCF-DA for 20 min and trypsinized with 1 × trypsin-EDTA at 37°C for 5 min. The cells were followed by centrifugation at 500 × g at 4°C for 5 min, and cell pellets were resuspended with ice-cold phosphate-buffered saline. ROS were detected and analyzed using a FACSort flow cytometer (Becton Dickinson, NJ, USA).

### Immunoblot detection of hyperoxidized 2-Cys PRXs

NCI-H460 and NCI-H1299 cells were seeded (1.5 × 10^5^) and subjected to various conditions for 48 h. NCI-H460 cells or NCI-H1299 cells were pre-treated with 10 or 30 μM AMRI-59 for 6 h and then irradiated 3 or 5 Gy IR, respectively. Following treatment for 48 h, cells were harvested, washed twice with ice-cold phosphate-buffered saline and lysed at 4°C in lysis buffer containing 20 mM HEPES-NaOH (pH 7.0), 2 mM EGTA, 1 mM EDTA, 1% Triton X-100, 1 mM AEBSF, aprotinin (10 μg/mL) and leupeptin (10 μg/mL) as described in a previous study [13]. Insoluble material was removed by centrifugation at 12,500×g for 10 min. Protein extracts were combined with sample loading buffer and immunoblot analysis performed using antibodies specific for 2-Cys PRX-SO_3._

### Mitochondrial potential (*ΔΨm*) detection assay

Cells were seeded at a density of 1.5 × 10^5^ and treated under various conditions. NCI-H460 cells or NCI-H1299 cells were pre-treated with 30 μM AMRI-59 for 6 h, and then irradiated 3 or 5 Gy IR, respectively. After IR irradiation, cells were incubated for 48 h. Incubated cells were treated with 10 μM JC-1 (Sigma-Aldrich) for 30 min and trypsinized with 1 × trypsin-EDTA at 37°C for 5 min. Cells were harvested by centrifugation at 500 × g at 4°C for 5 min, and the JC-1 -stained cells were analyzed using a FACSort flow cytometer (Becton Dickinson, NJ, USA). Increase in mitochondrial potentialwas monitored as a decrease in the fluorescence of JC-1 monomers (FL-1 channel).

### Measurement of caspase-3 activity

An ELISA kit purchased from Abcam (Cambridge, UK) was used to detect caspase-3 activity. NCI-H460 and NCI-H1299 cells were seeded onto a 100 mm plate (1 × 10^6^ cells/plate) and treated with control, 10 or 30 μM AMRI-59 or combinations of AMRI-59 and γ-IR for 72 h. After discarding the medium, cells were rinsed with PBS and dissolved in 1× cell lysate buffer provided in the kit. Supernatant fractions of sample lysates were collected via centrifugation at 500*g* for 5 min, followed by sequential mixture with reaction buffer and DEVD-*p*-NA substrate. Samples were incubated at 37°C for 60 min. The OD values at 405 nm were detected for each mixture in a Multiskan EX ELISA reader (Thermo Fisher Scientific, USA) and calculated as fold increase in caspase-3 activity between treated and untreated samples.

### Fractionation of mitochondria and cytosol

NCI-H460 and NCI-H1299 cells were treated with 30 μM AMRI-59 for 6 h, and then irradiated 3 or 5 Gy IR, respectively. After 24 h incubation, cells were harvested with 1 × Trypsin-EDTA, followed by centrifugation at 500 × g at 4°C for 5 min. Cell pellets were resuspended in extraction buffer (1 M sucrose, 1 M HEPES, pH 7.4, 1 M KCl, 1 M MgCl_2_, 0.25 M EGTA, and 1 M DTT) and homogenized. Homogenates were centrifuged at 14,000 × g at 4°C for 30 min. The supernatant was used as the cytosol fraction and the pellet resuspended in lysis buffer (150 mM NaCl, 50 mM Tris Cl pH 7.4, 0.1% Triton X-100, 1% NP-40, 0.25% sodium deoxycholate, 1 mM EGTA) as the mitochondrial fraction. Fractions were analyzed via immunoblot assay with a cytochrome c antibody.

### Immunocytochemical staining

NCI-H460 and NCI-H1299 (1 × 10^4^) cells were seeded in chamber slides and treated with 30 μM AMRI-59 for 24 h followed by 3 or 5 Gy IR. Treated cells were fixed with 1% paraformaldehyde and subsequently stained with a γ-H2AX antibody and DAPI. Images of stained cells were acquired under a LSM710 confocal microscope (Carl Zeiss, Germany).

### Propidium iodide uptake assay

Cells were seeded at a density of 1 × 10^5^ cells and incubated with or without 10 or 30 nM AMRI-59. After 16 h incubation, cells were exposed to 3 Gy IR and incubated for 48 h. Treated cells were harvested via trypsinization, washed twice with cold PBS, and resuspended in 300 μL of a 5 μg/mL propidium iodide (PI, Sigma-Aldrich) solution. The apoptotic fraction was evaluated using a FACSort flow cytometer (Becton Dickinson).

### Xenograft size determination and TUNEL assay

All animal experiments were performed using approved protocols of our Institutional Animal Care and Use Committee. The *in vivo* radiosensitization effects of AMRI-59 were evaluated in a xenograft model created by injecting NCI-H460 and NCI-H1299 cells subcutaneously into six week-old BALB/cAnNCrj-nu/nu mice (Envigo, Cambridgeshire, UK). Mice injected with these cells were divided into four groups (5 mice/group): control (mock treated), IR only, AMRI-59 only, and AMRI-59 and IR (combination treatment). When xenografts reached ∼100–120 mm^3^ in volume, AMRI-59 (100 mg/kg) was subcutaneously injected into tumor sites in mice in both AMRI-59-only and combination groups. Tumors in IR-only and control groups were injected with equal volumes of vehicle solution (DMSO). After 6 h, mice in IR-only and combination treatment groups were locally irradiated (3 or 5 Gy) using a ^60^Co γ-ray source (Theratrom 780; AECL Ltd., Mississauga, Ontario). This protocol was repeated four times at 3-day intervals for 10 days. Tumor dimensions (long and short axes) were measured periodically and tumor volumes calculated as (short axis^2^ × long axis/2). Based on tumor size analysis, growth delay values were calculated as described previously [42]. For the terminal deoxynucleotidyl transferase dUTP nick end labeling (TUNEL) assay, tumors were extracted, fixed with formaldehyde, and embedded in a paraffin block. Sliced tissues were stained and analyzed with the Apoptag TUNEL assay kit (EMD Millipore Co, CA, USA) as described by the manufacturer. Data from the TUNEL assay were quantified by counting the TUNEL-positive cells of each group. The number of TUNEL-positive cells of the control group was taken as the standard and then calculated as percentages from which percentages were calculated of each group [41].

### Statistical analysis

Data were analyzed using Graphpad Prism software (Graphpad Software, La Jolla, CA), and the significance of differences between experimental groups determined using Student *t*-test. *P*-values< 0.05 were considered significant. Individual *p*-values in figures are denoted by asterisks (^*^; *p* < 0.05, ^**^; *p* < 0.01, ^***^; *p* < 0.001). The numbers above each point or bar in the graphs represent the means of three independent experiments and error bars signify standard deviations (SD).

## SUPPLEMENTARY MATERIALS FIGURES


