# Supplementary material for: AMRI-59 functions as a radiosensitizer via peroxiredoxin I-targeted ROS accumulation and apoptotic cell death induction
Source: Oncotarget. 2017 Dec 9;8(69):114050–64. doi: 10.18632/oncotarget.23114 (PMC5768385; doi:10.18632/oncotarget.23114)
Supplement: Supplementary file 1 [file oncotarget-08-114050-s001.pdf]

AMRI-59 functions as a radiosensitizer via peroxiredoxin I-targeted ROS accumulation and apoptotic cell death induction

SUPPLEMENTARY MATERIALS

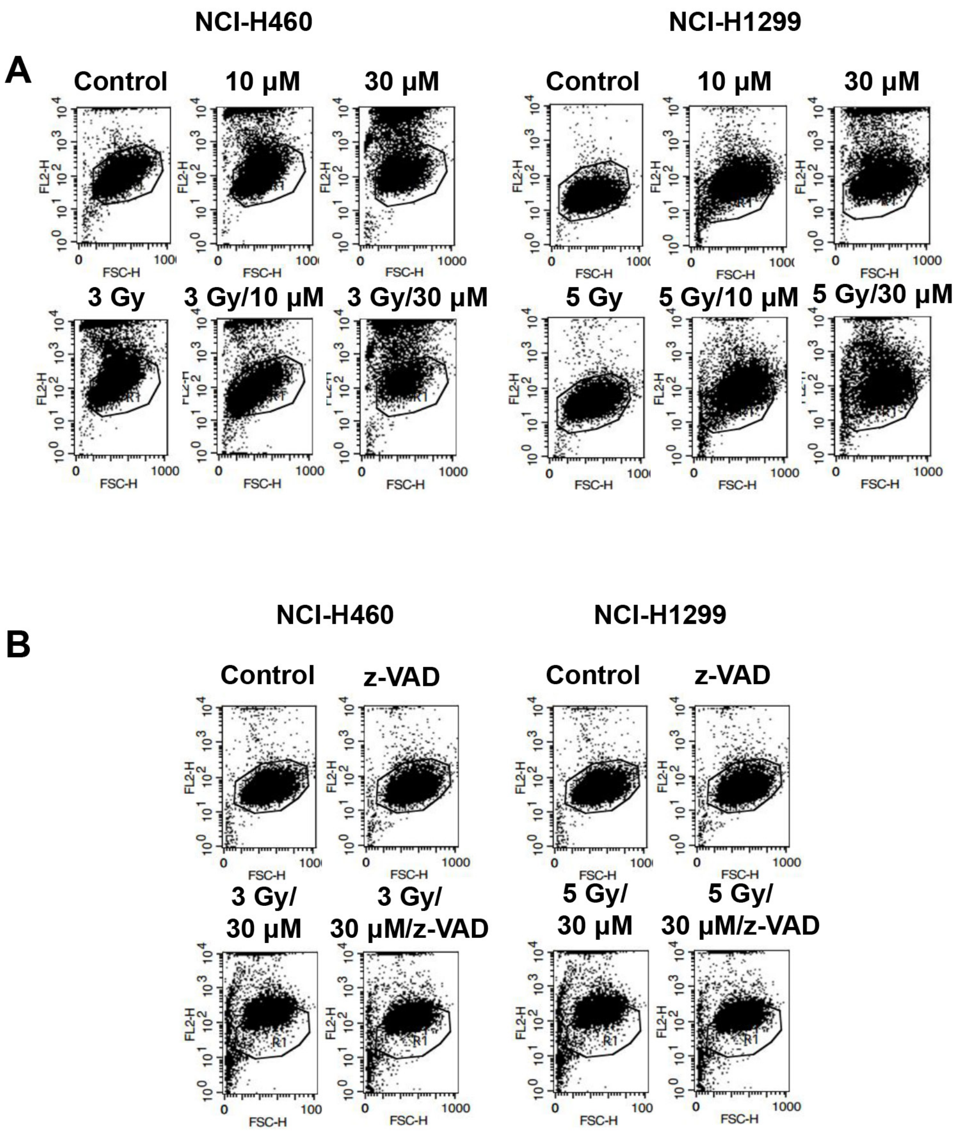

Supplementary Figure 1: AMRI-59 induces NSCLC cell death and enhances apoptosis in conjunction with IR. 'Control', mock control; '10 μM' and '30 μM', treatment with 10 and 30 μM AMRI-59 only; '3 Gy' and '5 Gy', treatment with 3 and 5 Gy IR only, respectively; '3 Gy/10 μM', '3 Gy/30 μM' and '5 Gy/10 μM', '5 Gy/30 μM', combination with 3 or 5 Gy IR and 10 or 30 μM AMRI-59, respectively; 'z-VAD', 20 μM of z-VAD-fmk pre-treatment for 1 h. **(A)** PI uptake assay for detection of apoptosis. NCI-H460 or NCI-H1299 cells were treated with various doses of AMRI-59 and IR. **(B)** PI uptake assay with or without pre-treatment of z-VAD-fmk for 1 h combined with AMRI-59 and IR.

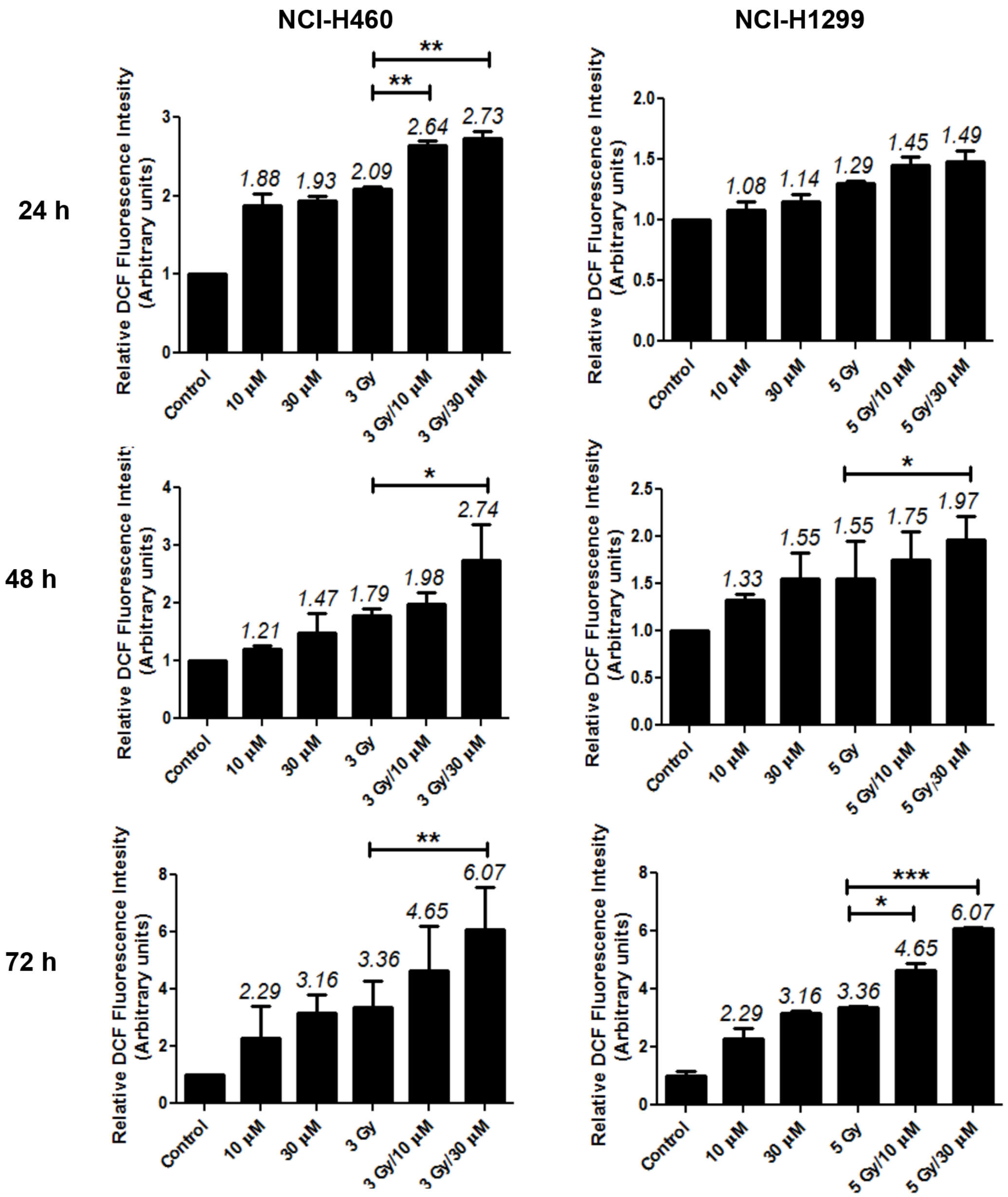

Supplementary Figure 2: AMRI-59 promotes ROS production in conjunction with IR. 'Control', mock control; '10 μM' and '30 μM', treatment with 10 and 30 μM AMRI-59 only; '3 Gy' and '5 Gy', treatment with 3 and 5 Gy IR only, respectively; '3 Gy/10 μM', '3 Gy/30 μM' and '5 Gy/10 μM', '5 Gy/30 μM', combination with 3 or 5 Gy IR and 10 or 30 μM AMRI-59, respectively. ROS detection with FACSorter in IR- only, AMRI-59 only- treated or combination of IR and AMRI-59 –treated NSCLC cells were performed in time-dependent manner at 24, 48 or 72 h.
